# The Effect of Gut Microbiome Perturbation on the Bioavailability of Glycyrrhizic Acid in Rats

**DOI:** 10.3390/pharmaceutics17040457

**Published:** 2025-04-01

**Authors:** Tiantian Shi, Huifang Li, Zihao Zhang, Yuying Zang, Shu Jiang, Tianjie Yuan

**Affiliations:** School of Pharmacy, Nanjing University of Chinese Medicine, No. 138 Xianlin Avenue, Nanjing 210023, China; 20220856@njucm.edu.cn (T.S.); 20200718@njucm.edu.cn (H.L.); 20220867@njucm.edu.cn (Z.Z.); 20230642@njucm.edu.cn (Y.Z.); jiangshu2000@163.com (S.J.)

**Keywords:** gut microbiome, probiotic, antibiotic, glycyrrhizic acid, bioavailability

## Abstract

**Background**: Oral administration remains the most common route for drug absorption. Emerging evidence highlights the important role of gut microbiome in the pharmacokinetics of oral medications. Glycyrrhizic acid (GL), a widely used hepatoprotective drug, is orally administrated and subsequently biotransformed by the gut microbiota into its active metabolite, glycyrrhetinic acid (GA), which exerts a therapeutic effect. However, it remains unclear whether the disturbance of the gut microbiome directly impacts the metabolism of GL. **Methods**: Antibiotic cocktail and probiotic *Lacticaseibacillus rhamnosus* R0011 were applied as two interventions targeting the gut microbiome. Pharmacokinetic parameters were evaluated by LC-MS, and 16S rRNA sequencing was applied to analyze the gut microbiome composition. The transcriptome analysis of Caco-2 cells was used to elucidate the regulation mechanism of polar metabolites resulting from gut microbiome perturbation. **Results**: R0011 supplementation could significantly increase the Area Under Curve (AUC) value of GA, which was positively correlated with the change in gut microbiome composition. In contrast, the plasma levels of GA were nearly undetectable following antibiotic intervention. Furthermore, the relative expressions of transporter multidrug resistance gene 1 (MDR1) in the ileum were site specifically downregulated under the probiotic intervention. The polar gut microbial metabolites may play a crucial role in differentiated regulating MDR1 expression, likely through the modulation of transcription factors FoxO1 and TP53. **Conclusions**: Our research provides new insights into the regulatory mechanism by which the gut microbiome affects the bioabsorption of orally administrated drugs, potentially offering strategies to optimize drug bioavailability and improve clinical efficacy.

## 1. Introduction

Oral administration remains one of the most common routes for drug delivery due to its convenience, cost-effectiveness, and safety [1]. Over the past decades, the pharmaceutical industry has made significant efforts to improve oral drug formulation, particularly to enhance solubility and intestinal permeability [2]. However, the gut microbiome—a complex community of symbiotic microbes—plays a crucial yet often overlooked role in modulating drug bioavailability. Emerging evidence indicates that gut microbiota can directly influence the pharmacokinetics and pharmacodynamics of orally administered drugs, thereby altering their therapeutic efficacy. Gut microbial nitroreductases can convert berberine (BBR) into its highly absorbable derivative, dihydroberberine (dhBBR), which exhibits a fivefold higher intestinal absorption rate than BBR itself [3]. On the other hand, there are a number of intestinal flora that could secrete β-glucuronidase [4], which effectively releases and reactivates glycosidins in the gastrointestinal tract, such as *Bacteroidetes* and *Firmicutes* [4,5]. Variations in β-glucuronidase activity can further impact drug disposition by modulating the enterohepatic recirculation of bioactive compounds.

Beyond direct interactions, the gut microbiome can indirectly influence oral drug bioavailability by modulating host metabolic pathways and transporter activity. For example, a pharmacokinetic study of the immunosuppressant tacrolimus (TAC) revealed that antibiotic-induced gut microbiota depletion reduced whole-blood TAC exposure by 33%. Further analysis indicated that the TAC plasma exposure was closely associated with intestinal microbiota metabolites, likely via regulating the relative expression of MDR1 to enhance TAC bioavailability [6]. The gut microbiota and its metabolites can also directly regulate transporter expression. For example, lipopolysaccharides (LPSs) derived from gut bacteria suppress vitamin C uptake by downregulating sodium-dependent vitamin C transporters (SVCT-1 and SVCT-2) [7]. Conversely, microbial metabolites such as short-chain fatty acids (SCFAs) and secondary bile acids have been shown to upregulate MDR1 expression and function both in vitro and in vivo [8]. Additionally, butyrate, a key SCFA, enhances breast cancer resistance protein (BCRP) activity via peroxisome proliferator-activated receptor gamma (PPARγ) activation [9]. Collectively, these findings underscore the gut microbiome’s critical role in determining the bioavailability of orally administered drugs. Consequently, perturbations in gut microbial composition—whether through antibiotics or probiotics—can significantly alter drug pharmacokinetics and clinical efficacy. For instance, antibiotic-induced clearance of the gut microbiome could reduce lovastatin absorption [10], while probiotic *Lactobacillus* supplementation modifies acetaminophen metabolism by reshaping the metabolic capacity of gut bacteria [11].

Glycyrrhizic acid (GL), a prominent bioactive compound derived from licorice, offers a range of beneficial properties, including acting as a sweetener, detoxifying agent, and therapeutic agent, particularly in relation to liver injury [12]. Structurally, GL consists of one molecule of glycyrrhetinic acid (GA) and two molecules of glucuronic acid [13]. However, its oral bioavailability is limited by high molecular weight and low gastrointestinal permeability [14]. The metabolic process of orally administrated GL in vivo is relatively well understood; the glucuronic acid moiety can be enzymatically hydrolyzed by gut microbial glucuronidase. This enzymatic conversion produces GA, which possesses enhanced membrane permeability and robust pharmacological activity. Following oral ingestion, GA is the primary metabolic form of GL that is absorbed into the blood and exerts therapeutic effects [15]. Consequently, the bioavailability of GL is closely linked to the gut microbiome.

Our previous work revealed that the probiotic *Lacticaseibacillus rhamnosus* R0011 (R0011) could modulate gut dysbiosis and enhance GL bioavailability under liver injury status. However, the broader impact of gut microbiome perturbation on oral GL metabolism, particularly indirect regulatory mechanisms, remains unexplored. In this study, antibiotic cocktail and probiotic supplementation (R0011) were applied in the gut microbiome intervention. We integrated both in vitro and in vivo approaches to explore whether disturbances in the gut microbiome could also influence the pharmacokinetics of hepatoprotective agent GL in rats. Our findings elucidate novel mechanisms by which the gut microbiome influences the bioabsorption of orally administered drugs, offering potential strategies to optimize bioavailability and clinical efficacy.

## 2. Materials and Methods

### 2.1. Animals

Male SD rats (SPF grade, 180–220 g), purchased from Qinglongshan Animal Breeding Center, Hangzhou City, Zhejiang Province, were randomly divided into 3 groups with 6 rats in each group and 2 cages in each group. The rats were kept at a constant temperature (24 ± 2 °C), constant humidity (45–65%), and the light-dark period was 12 h in the SPF rat feeding center of the Experimental Animal Center of Nanjing University of Chinese Medicine. Sterile water for animals is prepared by the Laboratory Animal Center. Before the experimental treatment, the animals acclimated to the feeding conditions for one week. Rats in the same cage received the same treatment for the same group to avoid confounding factors such as the coprophagous effect.

### 2.2. Antibiotic and R0011 Treatment

Starting from the second week, the normal group (CON) was given gavage of normal saline, while the R0011 group (LGG) was given gavage of 1 × 10^9^ CFU/mL R0011 suspended in normal saline (R0011 was obtained from China General Microbiological Culture Collection Center (CGMCC) and the entry number was 1.8882. Man–Rogosa–Sharpe (MRS) broth (Solarbio, Beijing, China) was cultured in an anaerobic environment (37 °C) for 24 h and then centrifuged at 8000 rpm at 4 °C for 10 min to collect bacteria. The sediment is washed twice and re-suspended in sterile saline (Yuanye, Shanghai, China) with a final concentration of 1 × 10^9^ CFU/mL for further use) [16]. In the antibiotic group (ATB), four mixed antibiotics (vancomycin 100 mg/kg, neomycin sulfate 200 mg/kg, metronidazole 200 mg/kg, ampicillin 200 mg/kg) were dissolved in sterile normal saline by gavage on time for one week [17].

### 2.3. Sample Collection

Normal saline, probiotics, or antibiotics were used for pretreatment for one week. After the last gavage, fresh feces were collected in a dry and sterile centrifuge tube and placed at −80 °C. A single dose of 50 mg/kg (5 mg/mL) GL (Yuanye, Shanghai, China) suspended in normal saline was administered to the rats by oral gavage after 12 h of fasting. After the administration of GL, blood was collected from the orbital venous plexus at different time points (0.17 h, 0.33 h, 0.5 h, 0.75 h, 1 h, 2 h, 4 h, 6 h, 8 h, 12 h, 24 h, 48 h, 72 h), and 0.5 mL blood was collected from each time point into 1.5 mL tubes containing heparin sodium. Plasma was separated by centrifugation at 6000 r/min for 10 min and refrigerated at −80 °C. All rats were euthanized, and the colon and ileum were washed with PBS (Biosharp, Hefei, China) and stored at −80 °C until further analysis. All experiments were conducted in accordance with the guidelines and approval of the Animal Experiment Ethics Committee of Nanjing University of Chinese Medicine (ACU180305).

### 2.4. Cell Culture

Human epithelial colorectal adenocarcinoma cells (Caco-2 cells, HTB-37 BSL 1, ATCC, Gaithersburg, MD, USA) were cultured in Dulbecco’s modified DMEM medium (Jiangsu Keygen, Nanjing, China), supplemented with 10% fetal (*v*/*v*) bovine serum (Gibco, San Francisco, CA, USA) and 1% (*v*/*v*) penicillin-streptomycin dual antibody, in a sterile constant temperature cell incubator at 37 °C, 5% CO_2_, and 95% wet air, and their cell status was observed daily using an inverted microscope. Replace culture medium periodically to maintain proper nutrient levels and pH balance. When Caco-2 cells reached 80–90% confluence, the subculture was carried out. The newly revived cells were subcultured for three times, and subsequent experiments were carried out.

### 2.5. Preparation of Fecal Water

Fresh feces (500 mg) were weighed in a centrifuge tube. After adding 1 mL sterile phosphate buffered saline (PBS), the test tube was fully swirled to obtain a uniform suspension. Then, in the ultracentrifuge at 4 °C, the centrifuge tube was centrifuged at 4500 g/min for 15 min. After ultracentrifugation, the supernatant was filtered through a 0.22 μm sterile filter to obtain sterile fecal water (FW) [18]. Finally, the supernatant is stored at −20 °C to treat the cells.

### 2.6. FW Cytotoxicity Determination

Caco-2 cells in the logarithmic growth stage were digested with pancreatic enzymes, the supernatant was discarded by complete medium suspension, and the cells were counted using blood cell counting plates. After the cells were diluted to 5 × 10^4^/mL in complete media, 100 μL of diluted cell suspension was added to each hole behind the 96-well plate, and the cells were cultured in a cell constant temperature incubator for 48 h. The culture medium was absorbed, and 200 μL of FW diluted by DMEM medium was added, 5, 10, and 20 times, respectively. After 48 h of treatment with FW, 20 μL CCK8 (Biosharp, Hefei, China) reagent was added, and the cell culture was incubated in the cell incubator for 3 h. The light absorption value at 450 nm was determined by a microplate reader (PerkinElmer, Waltham, MA, USA), and the cell survival rate was calculated.

### 2.7. Co-Incubation of Cells and FW

Caco-2 cells at the logarithmic growth stage were digested, centrifuged, and inoculated into 6 well plates at a density of 1 × 10^6^ cells/well. After 48 h of wall fixation, the medium was renewed and the medium was replaced by either the FW or PBS. After 48 h of exposure [6], cells were washed and collected for a subsequent Rhodamine 123 (Biosharp, Hefei, China) uptake assay and quantitative analysis of related transporters and transcriptome analysis. At this concentration, FW was not toxic to cells.

### 2.8. Determination of MDR1 Function in Cells

To evaluate the function of the transporter MDR1, cells collected from co-incubation with FW were washed twice with cold PBS, treated with 2 μM Rhodamine 123 (1%DMSO), incubated at 37 °C for 35 min, and washed with cold PBS to terminate the uptake reaction. The average fluorescence intensity of Caco-2 cells was analyzed by flow cytometry and inverted fluorescence microscopy to determine the effect of fecal water on the function of Caco-2 cells [19,20].

### 2.9. Real-Time Fluorescence Quantitative PCR

Total mRNA was extracted from colon, ileum tissues, and cells treated with FW by Free Reagent (Vazyme, Nanjing, China), and cDNA was obtained by reverse transcription of HiScript II Q RT SuperMix for qPCR (+gDNA wiper). Quantitative PCR was performed using ChamQ SYBR qPCR Master Mix (Without ROX) at 95 °C for 10 s and 60 °C for 30 s for 40 cycles. The relative gene expression was calculated by the 2^−ΔΔCt^ method with GAPDH as the reference gene. Primer sequences for the target genes are detailed in Table 1.

### 2.10. Pharmacokinetic Analysis of GA

The rat plasma collected and stored at −80 °C was thawed at room temperature, 100 μL plasma samples were taken at different time periods, and 10 μL of internal standard and 300 μL of methanol solution were added and vortexed for 2 min. After centrifugation at 13,000 r/min for 10 min, the supernatant was filtered to remove proteins from the plasma. A total of 200 µL was used for UPLC-TQ-MS/MS analysis. The analysis was performed on the ACQUITY UPLCBEH C18 column, 2.1 × 100 mm, 1.7 μM (Milford, MA, USA), and the column temperature was maintained at 40 °C. LC-MS/MS analysis was performed in conjunction with a Waters HPLC System (Milford, MA, USA) and a Waters Xevo TQD Triple Quadrupole Mass Spectrometer (Milford, MA, USA). The mobile phase consisted of (A) 0.4% FA in distilled water and (B) acetonitrile. The gradient procedure is used as follows: 0–1 min, 70–20% A; 1–3 min, 20% A; 3–3.5 min, 20–70% A; and 3.5–4.5 min, 70% A. The flow rate was 0.3 mL/min and the sample volume was 10 μL. The gas flow is 650 L/Hr; Spray voltages: 3100 V; ionization source: ESI-; Mode: MRM; standard curves: Y = 0.01586x + 0.00165 (R2 = 0.99770, 0.5~600 ng/mL); LOQ: 0.5 ng/mL, LOD: 0.2 ng/mL; Fragmentation pattern: GL: *m*/*z* 821.1→*m*/*z* 351.0, GA: *m*/*z* 471.3→*m*/*z* 371.1, IS (Glibenclamide): *m*/*z* 491.9→*m*/*z* 169.8. The original chromatograph and MS data are submitted in Supplementary Information (Figure S1).

### 2.11. Intestinal Flora Analysis

DNA was extracted according to E.Z.N.A.^®^ Soil DNA Kit instructions, and the integrity of DNA was tested using 1% agarose gel electrophoresis. PCR amplification was performed on primer pairs in the 338F (5′-ACTCCTACGGGAGGCAGCAG-3′) and 806R (5′-GGACTACHVGGGTWTCTAAT-3′) regions of the V3 and V4 bacteria. The library was constructed using the NEXTFLEX Rapid DNA-Seq Kit and paired with sequencing based on the Illumina MiSeq PE300 platform (Illumina, San Diego, CA, USA). Using UPARSE software (http://drive5.com/uparse/, version 7.1, accessed on 2 March 2023) [21], with 97% of the similarity to the sequence of operation taxa (OTUs) clustering analysis, will remove chimeric sequences. Species classification annotation was performed on each sequence using an RDP classifier (https://ngdc.cncb.ac.cn/databasecommons/database/id/237/, version 2.2, accessed on 2 March 2023) [22], the sequence was compared with the Silva 16S rRNA database (version 138), and the comparison threshold was set to 70% (Accession number: PRJNA1066995).

### 2.12. Cellular Eukaryotic Reference Transcriptome Analysis

RNA purification, reverse transcription, library construction, and sequencing were performed at Shanghai Majorbio Bio-pharm Biotechnology Co., Ltd. (Shanghai, China) according to the manufacturer’s instructions (Illumina, San Diego, CA, USA). The cell RNA-seq transcriptome library was prepared following Illumina^®^ Stranded mRNA Prep Ligation from Illumina (San Diego, CA, USA) using 1 μg of total RNA. Shortly, messenger RNA was isolated according to the polyA selection method by oligo(dT) beads and then fragmented by a fragmentation buffer first. Secondly, double-stranded cDNA was synthesized using a SuperScript double-stranded cDNA synthesis kit (Invitrogen, Carlsbad, CA, USA) with random hexamer primers (Illumina). Then, the synthesized cDNA was subjected to end-repair, phosphorylation, and ‘A’ base addition according to Illumina’s library construction protocol. Libraries were size-selected for cDNA target fragments of 300 bp on 2% Low Range Ultra Agarose followed by PCR amplified using Phusion DNA polymerase (NEB) for 15 PCR cycles. After being quantified by Qubit 4.0, the paired-end RNA-seq sequencing library was sequenced with the NovaSeq X Plus sequencer (2 × 150 bp read length). To identify DEGs (differential expression genes) between two different samples, the expression level of each transcript was calculated according to the transcripts per million reads (TPM) method (Accession number: PRJNA1077885).

### 2.13. Statistical Analysis

Data are shown as the mean ± SD. Statistical analysis was performed via GraphPad Prism 9 (GraphPad Software, San Diego, CA, USA, https://www.graphpad.com). All of the data were analyzed via Student’s *t*-test or post-hoc method ‘tukey’ for the ANOVA test. Except for the ANOVA test employed in alpha diversity, the Student’s *t*-test was utilized for all other statistical analyses. The details of the tests used are displayed in the figure legends. The threshold for statistical significance was set as *p* < 0.05. Spearman correlation analysis was used in the correlation analysis.

## 3. Results

### 3.1. Antibiotics and R0011 Differentially Impact the Metabolism of GL

**Figure 1:**
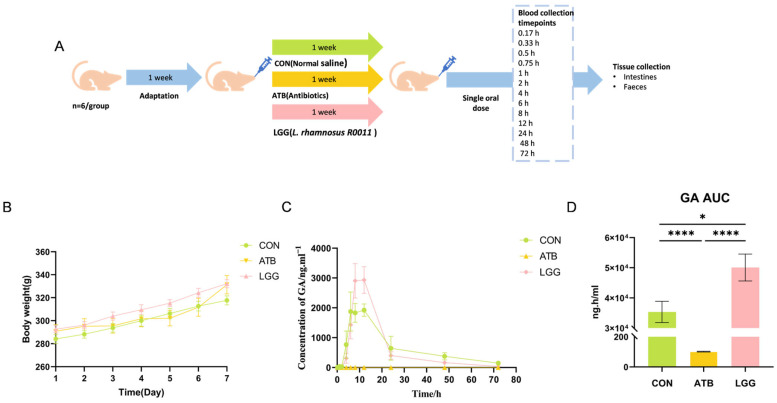
After treatment with *Lacticaseibacillus rhamnosus* R0011 (LGG) and antibiotics (ATB), the bioavailability of glycyrrhetinic acid (GA) was affected. (**A**) Experimental timeline. (**B**) Neither the use of probiotics nor the administration of antibiotics influenced body weight gain. (**C**) Blood concentration of GA in the control group, antibiotics, and LGG treatment group. (**D**) GA area under the curve (AUC). (**** *p* < 0.0001, * *p* < 0.05. *n* = 6 per group).

Following a week of dietary adaptation, the Sprague Dawley (SD) rats assigned to the LGG group received R0011 at a dosage of 1 × 10^9^ CFU/mL via oral gavage. Concurrently, the ATB group was treated with a gavage of antibiotics mixture, whereas the CON group was administered normal saline for the entire week (Figure 1A). The rat body weight was measured daily, and there were no significant differences observed among the three groups (Figure 1B).

GA is the primary metabolic form of GL that is absorbed into the blood and exerts therapeutic effects. Therefore, the pharmacokinetic parameters of GA were evaluated by LC-MS following the oral administration of GL. Our findings revealed that the plasma concentrations of GA changed significantly across all three groups (Figure 1C). The antibiotic-induced gut microbiota clearance leads to a remarkable reduction in GA levels at every time point. Compared to the CON group (AUC_0−t_ value= 35,345.7 ng·h/mL), the AUC_0−t_ value of the ATB group dramatically decreased to 101.9 ng·h/mL, with GA levels almost undetectable in the ATB group. In contrast, GA levels were notably elevated in the LGG group, and the AUC_0−t_ value reached 50,070 ng·h/mL (Figure 1D). Moreover, compared with the ATB group, Tmax/h and Cmax/ng·mL^−1^ of GA in the LGG group were significantly changed (Table 2). These results demonstrated that perturbations in the gut microbiome have significant effects on GL metabolism.

### 3.2. Antibiotic and R0011 Preconditioning-Altered Intestinal Flora

**Figure 2:**
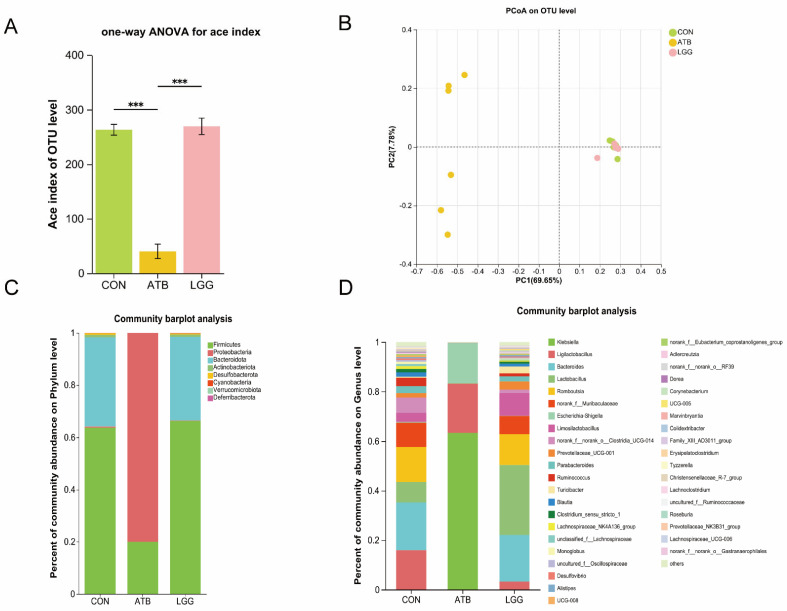
Changes in the intestinal flora composition of rats after pretreatment with antibiotics and *Lacticaseibacillus rhamnosus* R0011. (**A**) Alpha diversity estimators calculated indexes of Ace on the OTU level. (**B**) PCoA analyses were performed on the OTU level. (**C**) Phylum level species composition. (**D**) Genus level species composition. (*** *p* < 0.001. *n* = 6 per group).

To assess the impact of R0011 and antibiotic preconditioning on the gut microbiota, 16S rRNA sequencing of gut bacteria was conducted. The Chao1 index was significantly decreased in the ATB group compared to the LGG group, indicating a reduction in the diversity of intestinal microbes. Conversely, R0011 could enhance the richness of the intestinal flora in rats (Figure 2A). Additionally, principal coordinate analysis (PCoA) revealed significant β diversity differences between the antibiotic-treated group and the control group, as shown in the scatterplot, which suggests a substantial alteration in the microbial structure and composition of the rats in the ATB group (Figure 2B).

The gut microbial diversity analysis revealed notable disparities at both the Genus and Phylum levels when comparing the ATB group against the LGG group. In the LGG groups, the intestinal tracts of rats predominantly harbored *Firmicutes*, *Bacteroides*, and *Actinobacteriota* identified as the most abundant gut bacterial groups. In contrast, the ATB group experienced a significant shift due to antibiotic treatment, leading to a notable depletion of several genera and a predominance of *Firmicutes* and *Proteobacteria*. (Figure 2C). At the Genus level, the ATB group’s microbial community was predominantly characterized by Gram-negative bacteria such as *Klebsiella* and conditionally pathogenic bacteria like *Escherichia-Shigella*. The LGG group exhibited a richer diversity, with a substantial presence of *Lactobacillus*, *Ruminococcus*, and *Prevotellaceae* species (Figure 2D).

### 3.3. The AUC Value of GA Correlated with the Change in Gut Microbiome Composition

**Figure 3:**
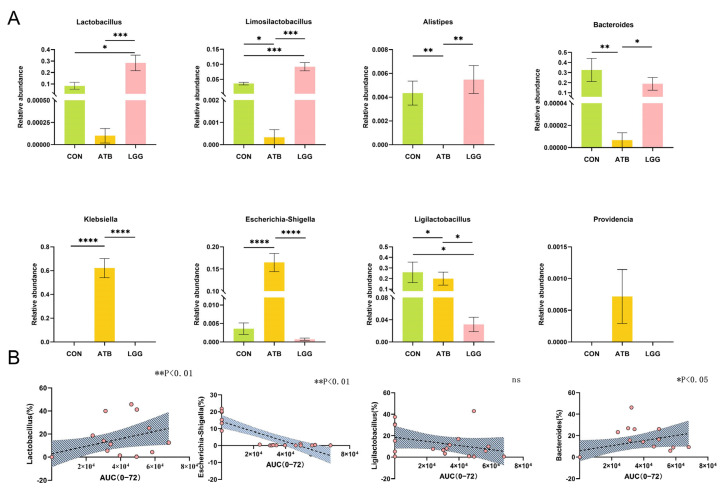
Spearman correlation between AUC and the relative abundance of glycosidase-containing bacteria in three groups. (**A**) The strain abundance in different treatment groups was expressed as the mean ± SEM (*n* = 6). (**B**) Relationship between *Lactobacillus*, *Escherichia-Shigella*, *Ligilactobacillus*, and *Bacteroides* and AUC of GA. The *x* axis of the orange dot is the AUC of glycyrrhetinic acid and the Y axis is the abundance of the Genus, trend curve of correlation between AUC of glycyrrhetinic acid and abundance of bacteria in different groups. (**** *p* < 0.0001, *** *p* < 0.001, ** *p* < 0.01, * *p* < 0.05. *n* = 6 per group).

A comparative analysis at the genus level revealed that certain bacterial groups, such as *Lactobacillus* and *Bacteroides*, were significantly enriched in the LGG groups. Conversely, *Escherichia-Shigella* was found to be significantly enriched in the ATB group (Figure 3A). Our results showed that the change in the pharmacokinetics of GA occurred in parallel with alterations in the gut microbiome. Therefore, we further investigated the correlation between the relative abundance of these specific bacterial taxa and the AUC value of GA.

Our findings indicated a positive correlation between the relative abundance of *Lactobacillus* and *Bacteroides* and the AUC value of GA. On the other hand, the relative abundance of *Escherichia-Shigella* exhibited a negative correlation with the AUC value of GA, with a statistically significant association (Figure 3B). *Ligilactobacillus*, which is abundant in both the LGG and CON groups, showed no correlation with the AUC value of GA.

### 3.4. The Expression of MDR1 in the Ileum Decreased Under R0011 Intervention

**Figure 4:**
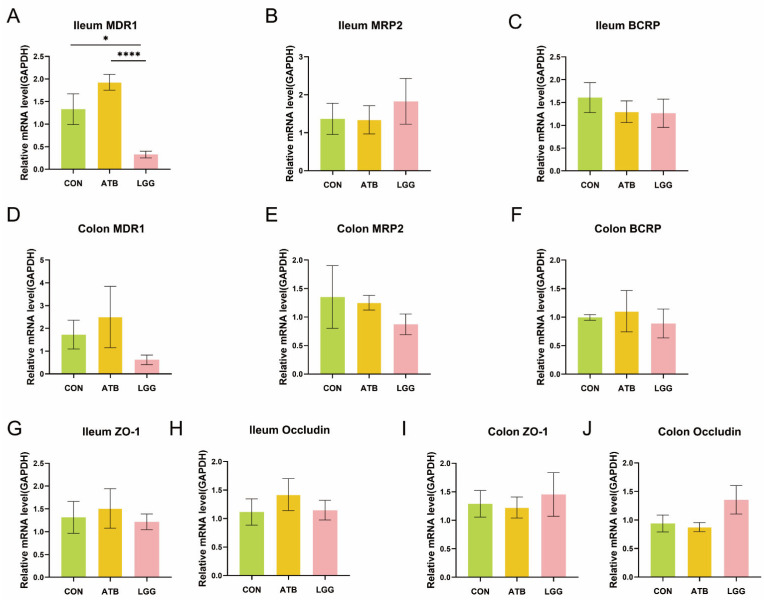
Expression of transporters in. the ileum and colon. (**A**–**C**) To compare the mRNA expression of efflux transporters MDR1 (**A**), MRP2 (**B**), and BCRP (**C**) in the ileum of different treatment groups. (**D**–**F**) To compare the mRNA expression of efflux transporters MDR1 (**D**), MRP2 (**E**), and BCRP (**F**) in the colon of different treatment groups. (**G**–**J**) To compare the mRNA expression of the tight junction protein in the ileum (**G**,**H**) and colon (**I**,**J**) of different treatment groups. (**** *p* < 0.0001, * *p* < 0.05. *n* = 6 per group).

Drug transporters are pivotal determinants of the bioavailability of orally administered medication, and GA is the natural substrate of intestinal efflux pumps. Therefore, an analysis of the expression levels of relevant transporters in the colon and ileum was conducted. It was observed that pre-treatment with R0011 significantly reduced the expression of the efflux transporter MDR1 in the ileum, a notable difference when compared to the ATB group (Figure 4A). However, this pre-treatment did not significantly alter the expression levels in the colon (Figure 4D). The efflux transporters BCRP and MRP2 in both the ileum and colon showed no significant differences across the three groups (Figure 4B,C,E,F). Additionally, it is essential to note that the host’s intestinal barrier also plays a crucial role in bioactive compound absorption. To rule out the possibility of an increase in GA absorption due to perturbation-induced damage to the intestinal barrier, we assessed the relative expression of tight junction proteins ZO-1 and Occludin, which are integral to maintaining the intestinal barrier in the ileum and colon in rats. The findings indicated that neither antibiotics nor R0011 had any significant effect on the integrity of the intestinal barrier (Figure 4G–J).

### 3.5. Gut Microbial Metabolites Affect MDR1 Expression but There Is No Direct Impact on Function

The intestinal microbiota is recognized as a significant source of metabolic compounds and plays a key role in regulating various physiological processes. To further understand the regulatory mechanism by which gut microbiome influences the bioabsorption of GA, we speculated that the expression and function of intestinal MDR1 may be influenced by gut microbial metabolites. Therefore, we conducted an experiment in which Caco-2 cells were exposed to fecal water (FW) to assess the regulatory role of intestinal metabolites on the expression and function of efflux transporters.

**Figure 5:**
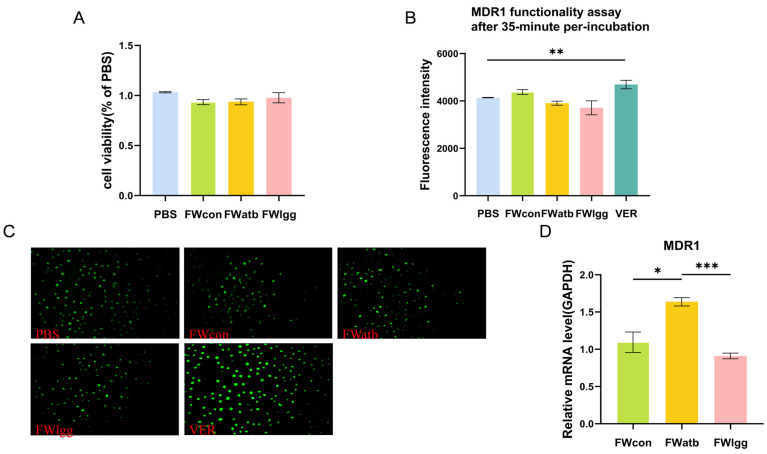
Effects of fecal metabolites on Caco-2 cell transporters. (**A**) Fecal water cytotoxicity cck8 assay. (**B**,**C**) MDR1 substrate (rhodamine 123) intracellular accumulation with or without a 35 min pre-exposition to mouse fecal water (FW) for fluorescence intensity (**B**) and microscopic images (200 µm) (**C**). (**D**) Comparison of the mRNA expression of MDR1 in control Caco-2 cells (PBS) or cells exposed for 48 h to FW. (*** *p* < 0.001, ** *p* < 0.01, * *p* < 0.05. *n* = 6 per group).

Firstly, we confirmed that the concentration of FW used in the experiment was non-toxic to the cells (Figure 5A). Subsequently, we examined the impact of FW on the transport function of MDR1. The cells were treated with the fermentation water (FW) from the CON, LGG, and ATB groups, and the fluorescence intensity of Rhodamine 123, a substrate for the MDR1 transporter, was subsequently measured. Verapamil, a known MDR1 substrate, was used as a positive control (Figure 5B,C). The findings indicated that there were no significant differences in the fluorescence intensity among the groups. Moreover, we investigated the effects of microbial-derived metabolites on the relative expression of MDR1. Our results showed that the metabolites of supplementary R0011 led to a significant decrease in MDR1 expression compared to the ATB group (*p* < 0.05) and a moderate decrease compared to the CON group (ns) (Figure 5D). These data suggest that the changes in MDR1 expression were regulated by polar gut microbial metabolites, which have no direct effect on MDR1 efflux function.

### 3.6. Gut Microbial Metabolites Regulate MDR1 Expression by Transcriptional Regulators

**Figure 6:**
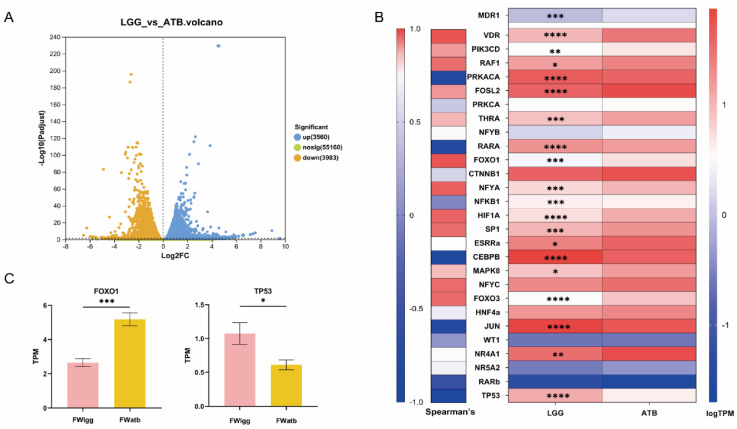
Effects of the microbiome on MDR1-associated transcription factors, transcriptomic analysis of Caco-2 cells exposed to FWatb and FWlgg. (**A**) Expression difference volcano map. (**B**) Analysis of transcription factors (TFS) known to induce, variable, or inhibit MDR1 expression. The left side shows the Spearman correlation between ABCB1 expression and each TF. On the right is a heat map showing the relative expression of relevant transcription factors in the FWlgg group compared to the FWatb group. (**C**) Expression levels of transcription factors FoxO1 and TP53 in the two groups. (**** *p* < 0.0001, *** *p* < 0.001, ** *p* < 0.01, * *p* < 0.05. *n* = 6 per group).

To elucidate the regulation mechanism of polar metabolites resulting from gut microbiome perturbation, we subjected Caco-2 cells in the logarithmic growth phase to FW from both the LGG and ATB groups. The transcriptome analysis was conducted and the results revealed significant differences in gene expression between the two groups: 3560 genes were up-regulated in the FWlgg group relative to the FWatb group and 3983 genes were down-regulated (Figure 6A). Based on the reported MDR1 transcriptional factors, including inducers, inhibitors, and controversial transcription factors [6,23], the correlation between transcription factor expression and MDR1 mRNA level was further analyzed.

We compared the relative expression profiles of FWatb and FWlgg (Figure 6B). The forkhead box-containing protein O subfamily1 (FoxO1) showed the strongest positive correlation with MDR1 expression and Cellular tumor antigen TP53 exhibited the strongest negative correlations (Figure 6C); our results showed that the relative expression of inducer FoxO1 was significantly downregulated in the LGG group compared with the ATB group, while the relative expression of inhibitor TP53 was evidently upregulated.

## 4. Discussion

Orally administered medications inevitably interact with the gut microbiome in vivo during the metabolism step, through direct or indirect mechanisms. These interactions significantly influence the drug bioavailability and alter the therapeutic outcomes. The gut microbiome could directly modify the drug structure by diverse enzymatic biotransformation pathways. Meanwhile, the gut microbiome could also produce microbial metabolites that act as signal molecules, potentially activating or inhibiting host gene transcription, which ultimately affects the drug’s metabolic fate. Therefore, understanding how the gut microbiome affects drug pharmacokinetics (PKs) and pharmacodynamics (PDs) is particularly important in the pharmaceutical industry [24].

In this study, GL was selected as the model for orally administrated drugs, which was widely used in clinical practice due to its hepatoprotective and antinflammation properties. GL, when orally administrated, is enzymatically hydrolyzed by gut microbial glucuronidase, allowing the metabolic GA to be absorbed into the bloodstream and exert a therapeutic effect. Therefore, the gut microbiome plays a vital role in GL metabolism. Nowadays, probiotics and antibiotics are commonly available in our daily lives, which potentially influence the composition and function of the gut microbiome [25]. However, the effect of these two types of intervention on the bioavailability of oral drugs has been underestimated. Our results revealed that plasma levels of GA were nearly undetectable following antibiotic intervention; however, probiotic supplementation could significantly increase GA bioabsorption, especially compared to the antibiotic group.

Supplementation with probiotic R0011 for one week resulted in a significant enrichment of *Bacteroidetes* and *Firmicutes* in the intestinal flora, which is consistent with the previously reported results [26]. However, following antibiotic treatment, pathogenic gram-negative bacteria including *Klebsiella* and *Escherichia-Shigella* dominated in the rats [27]. We speculated that the intestinal strains capable of expressing β-glucuronidase were largely eliminated, contributing to the reduced AUC value of GA in the ATB group. Our results also showed that the AUC value of GA was positively correlated with *Lactobacillus* and *Bacteroides,* which were enriched in the LGG group. These gut bacteria are known to harbor abundant glycosidase, which facilitates the bioconversion of GA. Additionally, our study suggested that chronic antibiotics could directly alter the composition of the gut microbiome, significantly impacting the metabolic fate of the oral drug. Previous research has reported a 1.8-fold increase in the bioavailability of olanzapine in rats subjected to antibiotic-induced depletion of gut microbiota [28]. In contrast, the bioabsorption of GL was nearly interrupted by antibiotic intervention. The clinical significance of antibiotics on oral drug metabolism may depend on the specific chemical properties of oral drugs and individual patient-related factors.

Furthermore, the gut microbiome has the potential to influence the host gene expression; our results indicated that probiotic intervention could significantly downregulate the relative expression of MDR1 compared to both the control and ATB groups, particularly in the ileum part. However, the perturbation of the gut microbiome has no effect on the gene expression of other transporter proteins as well as the tight junction proteins. Therefore, in addition to changes in gut microbiome composition, the increase in the AUC value of GA could be also closely related to MDR1 function and expression. It is well known that efflux transporters play a crucial role in drug bioabsorption; moreover, GA has been identified as a substrate for the intestinal efflux pump MDR1. It has been reported that certain small molecules can affect the expression of efflux transporters. For example, the short-chain fatty acid butyrate may significantly down-regulate P-gp (MDR1) expression and function by inhibiting the HDAC/NF-κB pathway [9]. Therefore, we speculated that the polar bacterial metabolite resulting from the probiotic and antibiotic intervention may site-specifically affect the efflux relative expression. We treated Caco-2 cells with fecal water prepared from different rat feces groups, simulating the microbial environment and metabolites in vitro to study their effects on Caco-2 cell transporters. Our results demonstrated that polar bacterial metabolites could not change the transport profile of Rhodamine 123, ruling out a direct impact on MDR1 function. However, the metabolites from probiotic intervention significantly downregulate the MDR1 expression compared with those from antibiotic treatment, suggesting that these gut microbial metabolites could differently module MDR1 transcriptional expression.

The possible mechanism of intestinal metabolites from perturbation affecting efflux transporters was further investigated by transcriptome analysis. The transcription factors related to the transporter MDR1 were previously identified by Chen et al. [29]. and Foley et al. [23]. In our study, we found that FoxO1 showed the strongest significant positive correlation with MDR1, whereas TP53 showed a pronounced negative correlation. It has been reported to reduce the mRNA and protein levels of MDR1 in human hepatocellular carcinoma cell line HepG2 by down-regulating the human ABCA1 gene involved in multiple transcription factors, such as FOXO1 [30]. Wang et al. demonstrated that the inhibition of the degradation of TP53 could lead to the upregulation of MDR1 [31]. While our experiment offered preliminary insights into the mechanism of metabolites affecting transporters, the specific bacterial metabolites are worthy of further analysis in subsequent exploration. Furthermore, it is essential to note that microbiome regulation of MDR1 expression cannot be limited to TP53 and FoxO1. Among other important TFS, expression profiles of VDR, RAF1, NFYA, and HIF1A also showed strong positive associations with MDR1, even if these associations were not as strong as those observed with FoxO1. Besides the transcription factors, the other genes upregulated or downregulated may also be involved in the regulation of MDR1 expression, indicating the need for further research to elucidate the underlying molecular mechanisms.

## 5. Conclusions

In our study, two gut microbiota targeting interventions were applied to assess the effect of the gut microbiome on GL metabolism. Our results showed that probiotic R0011 supplementation could significantly increase the AUC value of GA, which was positively correlated with the composition of the gut microbiome. Furthermore, the gut microbiome could influence GA bioabsorption by downregulating the relative expression of the efflux transporter MDR1 in the ileum. The gut microbiome-derived metabolites play a crucial role in this regulation of MDR1 expression, likely through the modulation of transcription factors Foxo1 and TP53, which are associated with the transporter MDR1.

## Figures and Tables

**Table 1:** Sequence of the primers used in this study.

Genes	Forward Primer (5′-3′)	Reverse Primer (5′-3′)
*MDR1*	GAGCCCATCCTGTTTGACTG	TGTCTCCCACTCTGGTGTTG
*MRP2*	TTCTGGATCCTCTCGGTCTTATG	ATCTGGAAACCGTAGGAGACGAA
*BCRP*	CAATGGGATCATGAAACCTG	GAGGCTGATGAATGGAGAA
*ZO-1*	CTGATGGTGTTCTGCCAAATTC	GTCGCAAACCCACACTATCT
*Occludin*	CCATCTGACTATGCGGAAAGAG	TACCAGAGGCGGTGACTTAT
Human Genes *MDR1*	CACCACTGGAGCATTGACTACC	TTGCCAACCATAGATGAAGGAT

**Table 2:** Pharmacokinetic parameters of GA under antibiotic and probiotic intervention.

Pharmacokinetic Parameters	CON	ATB	LGG
T_max_/h	8.67 ± 2.73	2.33 ± 4.75 *	10.67 ± 2.07
C_max_/ng·mL^−1^	3119.67 ± 1611.8	3.17 ± 2.84 ***	3670.93 ± 1038.73
AUC_(0−t)_/ng·mL^−1^·h	35,345.7 ± 8638.0	101.91 ± 5.90 ***	50,070.3 ± 10,933.0 *
MRT_(0−t)_/h	17.89 ± 6.12	35.4 ± 0.990 ***	16.07 ± 3.95
CL (mL^−1^·h^−1^·kg)	1191.41 ± 524.99	87,419.27 ± 64,090.95 ***	1123.34 ± 348.22

Compared to the normal control group, * *p* < 0.05, *** *p* < 0.001.

## Data Availability

The original contributions presented in this study are included in the article. Further inquiries can be directed to the corresponding author.

## Supplementary Materials

The following supporting information can be downloaded at: https://www.mdpi.com/article/10.3390/pharmaceutics17040457/s1, Figure S1: Selective ion chromatogram of glycyrrhizic acid and glycyrrhetinic acid in rat plasma: (A) blank plasma; (B) blank plasma with standard substance; (C) Plasma sample after oral administration of glycyrrhizic acid in rat.
