# Supplementary material for: The Effect of Gut Microbiome Perturbation on the Bioavailability of Glycyrrhizic Acid in Rats
Source: Pharmaceutics. 2025 Apr 1;17(4):457. doi: 10.3390/pharmaceutics17040457 (PMC12030048; doi:10.3390/pharmaceutics17040457)
Supplement: Supplementary file 1 [file pharmaceutics-17-00457-s001.zip › pharmaceutics-3501999-supplementary.pdf]

The Effect of Gut Microbiome Perturbation on the Bioavailability of Glycyrrhizic Acid in Rats

Tiantian Shi, Huifang Li, Zihao Zhang, Yuying Zang, Shu Jiang and Tianjie Yuan *

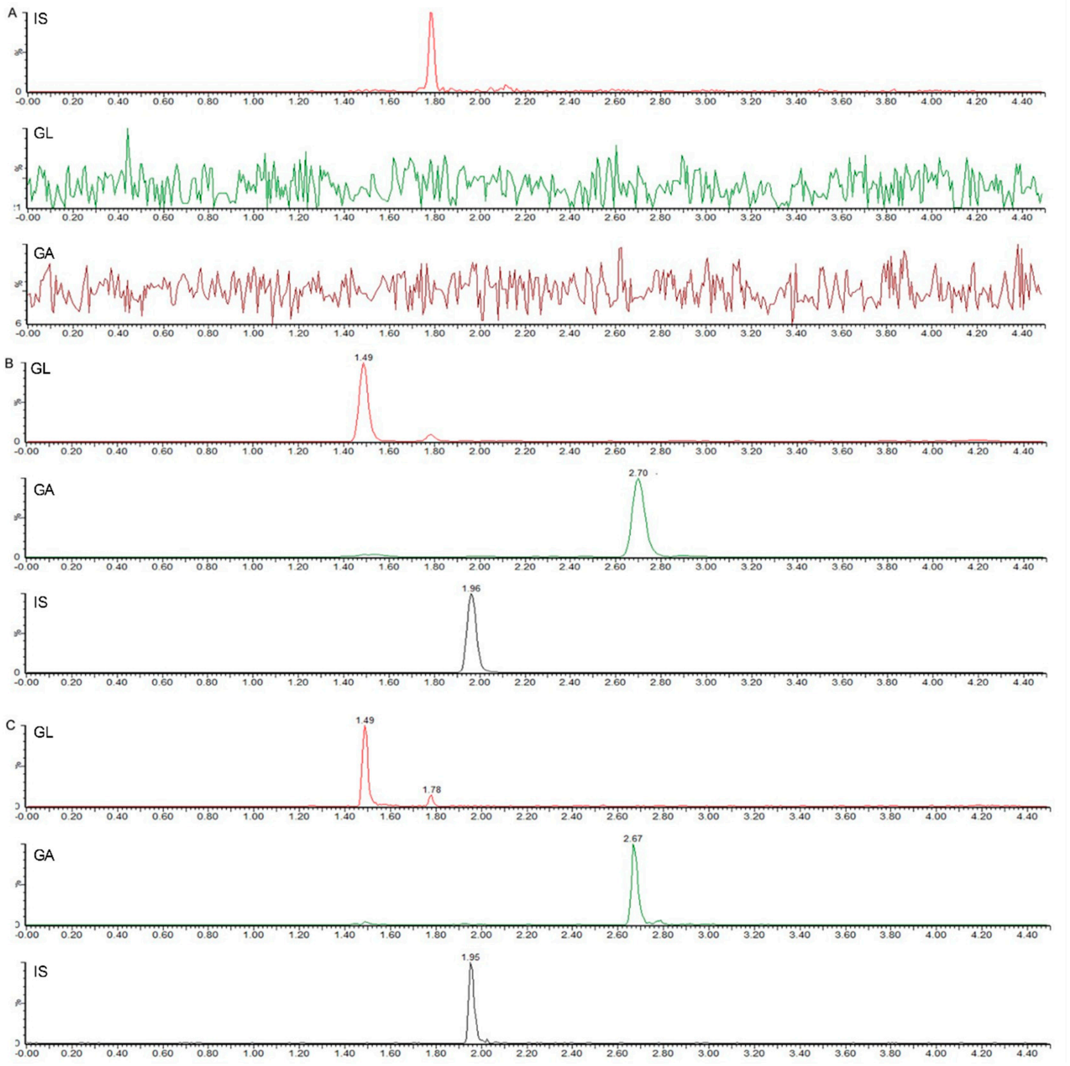

Figure S1. Selective ion chromatogram of glycyrrhizic acid and glycyrrhetinic acid in rat plasma (**A**) blank plasma; (**B**) blank plasma with standard substance; (**C**) Plasma sample after oral administration of glycyrrhizic acid in rat.
